# Quality of life as measured by the short-form 36 (SF-36) questionnaire in patients with early systemic sclerosis and undifferentiated connective tissue disease

**DOI:** 10.1186/1477-7525-11-23

**Published:** 2013-02-25

**Authors:** Michele Iudici, Giovanna Cuomo, Serena Vettori, Manuela Avellino, Gabriele Valentini

**Affiliations:** 1 Rheumatology Unit of the Second University of Naples, Via S. Pansini 5, Edificio 3, Naples 80131, Italy

**Keywords:** Quality of life, Systemic sclerosis, Undifferentiated connective tissue disease

## Abstract

**Objective:**

To investigate health-related quality of life (HRQOL) in patients affected by early systemic sclerosis (eSSc) and to compare it with that of patients with undifferentiated connective tissue disease (UCTD).

**Methods:**

At baseline, 31 eSSc and 35 UCTD patients underwent clinical evaluation, laboratory investigations, nailfold videocapillaroscopy, echocardiography, and lung function tests. All patients and 40 controls, matched for sex and age completed the Short Form-36 (SF-36) questionnaire and the Health Assessment Questionnaire Disability Index (HAQ-DI).

**Results:**

SF-36 scores were significantly lower in eSSc and UCTD patients than in healthy controls as regards the following domains: physical component score (PCS), mental component score (MCS), physical functioning, role-physical, bodily pain, general health and mental health. PCS was negatively correlated to the HAQ-DI (rho −0.59; p = 0.0004) and ESR >20 mm/h (rho −0.58; p = 0.0006) in eSSc patients. No statistically significant correlation was found between PCS, MCS and HAQ-DI in UCTD patients. Age, sex, disease duration, history of arthritis, low levels of either C3 or C4, a low DLCO (carbon monoxide lung diffusion) and inversion of the E/A ratio were not correlated to PCS and MCS in either eSSc or UCTD patients.

**Conclusion:**

Many eSSc or UCTD patients perceive they have an impaired quality of life in both physical and mental domains. This condition has to be taken into account by the clinicians involved in the care of these patients.

## Background

Systemic sclerosis (SSc) is an autoimmune rheumatic disease of unknown etiology characterized by microvascular injury and deposition of collagen and other constituents of extracellular matrix in target organs [1]. It is associated with a reduced life expectancy [2], significant functional disability [3-5] and depressive symptoms [6]. Health-related quality of life (HRQOL), as assessed by the Short Form-36 questionnaire (SF-36), is reduced in SSc patients compared both with the general population and with patients affected by such other common chronic conditions as heart and lung diseases, diabetes and arterial hypertension [7]. Previous studies that investigated HRQOL in patients with SSc were based on patients who fulfilled either the American College of Rheumatology (ACR) criteria for SSc [8] or LeRoy et al.’s criteria [9] or both. They revealed that HRQOL is impaired in patients suffering from each of the 2 main SSc subsets, namely, limited cutaneous (lc) SSc and diffuse cutaneous (dc) SSc, but that impairment is more pronounced in patients with dcSSc i.e. the subset characterized by a greater extent of skin sclerosis and earlier and more severe involvement of target internal organs [9].

Several years ago, Koenig et al. [10] reported that a high percentage of patients with Raynaud’s phenomenon (RaP) and an SSc marker autoantibody and/or typical SSc capillaroscopic findings and no manifestation other than puffy fingers and/or arthritis, develop definite SSc over time and proposed this condition be labeled “early (e)SSc”. These patients, by definition, have no clinical manifestation other than RaP. Thus far, no study has evaluated HRQOL in patients with RaP and eSSc.

The aim of the present study was to investigate HRQOL in patients affected by eSSc and to compare it with that of patients with undifferentiated connective tissue disease (UCTD) presenting only RaP and no other symptoms and that of healthy controls to determine whether the burden of secondary RaP and diagnosis of either eSSc or UCTD affects quality of life.

## Methods

### Inclusion/exclusion criteria

Patients consecutively admitted to the outpatient clinic of the Rheumatology Unit of the Second University of Naples for the evaluation of RaP from January 1st 2007 to December 31st 2011 were enrolled in the study if they fulfilled the following criteria: 1) Koenig et al.’s criteria for early SSc [10] (i.e., RaP with a marker autoantibody and/or a capillaroscopic scleroderma pattern without any clinical manifestation other than RaP, puffy fingers and/or present or past arthralgia/arthritis), or 2) Doria et al.’s criteria [11] for UCTD (i.e., they had serum ANA [antinuclear antibodies], but no marker autoantibody of SSc or specific of any other connective tissue disease, no scleroderma videocapillaroscopic findings or any clinical manifestation pathognomonic of any other connective tissue disease).

Patients fulfilling the American College of Rheumatology criteria for SSc [8] and those presenting at least one of the features of definite SSc according to Koenig et al. [10] were excluded. The diagnosis of RaP was confirmed if patients fulfilled LeRoy and Medsger’s criteria [9] i.e., if a cold challenge induced bilateral, episodic bi- or triphasic (pallor followed by dusky blueness and/or redness) color changes of fingers. The study was approved by the ethics committee of the Azienda Ospedaliera Universitaria, Seconda Università di Napoli. All patients agreed to participate to the study and provided written informed consent.

### Assessment of the patients

According to standard clinical practice and to ensure a correct classification, all patients underwent: a detailed history taking and a physical examination to identify the above-listed exclusion features, and puffy fingers and present or previous arthritis; routine laboratory investigations including cell blood count, urinalysis, BUN (blood urea nitrogen), ALT (alanine transaminase), AST (aspartate transaminase), erythrocyte sedimentation rate (ESR), serum protein electrophoresis and serum C3 and C4 concentration; widefield nailfold videocapillaroscopy (NVC) was carried out as described elsewhere [12] using a videocapillaroscope (Videocap 25-DS Medica-Italy) with optical probes of 200X. Stored images were reviewed by a physician (MI) experienced in NVC [13]. The degree of capillary enlargement on a scale of 0–3 and capillary loss (graded A–D) were investigated. Megacapillaries (capillary enlargement ≥2) and/or avascular areas (capillary loss grade ≥ C) were considered a scleroderma pattern [14,15]; a serum autoantibody profile, carried out as described elsewhere [12] on sera collected at the first visit, stored at −20°C and heated at 56°C for 30 min before testing, and including ANA, SSc and other connective tissue disease marker autoantibodies. ANA were considered positive when a diluition ≥ of 1:160 was registered, in absence of other known conditions associated with ANA positivity (i.e. chronic B or C hepatitis infection, autoimmune thyroid and hepatic disorders, malignancies). Thorax X-ray, barium esophageal X-ray and electrocardiogram were performed to identify patients that should be excluded from the study because of findings consistent with lung, esophageal or cardiac SSc involvement. Moreover, at baseline patients underwent B-mode echocardiography and lung function tests, performed as described elsewhere [16,17].

### Self-assessment questionnaires

Each patient and 40 healthy subjects recruited from the administrative, nursing and medical staff of the Department of Internal Medicine and matched for sex and age with the enrolled patients, after giving a written informed consent, were asked to complete self-administered questionnaires namely the Italian version of SF-36 [18] and the Health Assessment Questionnaire-Disability Index (HAQ-DI) [19]. The Italian version of SF-36 [18] consists of 36 questions grouped into 8 domains physical functioning, social functioning, role limitations related to physical problems, role limitations related to emotional problems, mental health, vitality, bodily pain and general health perception. A score ranging from 0 (indicating the worse health status) to 100 (indicating the best health status) is assigned for each domain. Domain scores can be summarized into a Physical Component Score (PCS) and Mental Component Score (MCS) [18]. For these two summary scales, a score below 50 reflects a worse HRQOL compared to the average of the general population [18]. HAQ-DI is a disease-specific, musculoskeletal-targeted questionnaire designed to assess functional ability in rheumatoid arthritis, the Italian version of which has been validated [19]; it has been extensively used and validated in SSc patients [3,4]. This questionnaire contains 20 items (each scored from 0 to 3) divided into 8 domains. The highest scores in each domain are summed and then divided by 8 to obtain the final HAQ-DI value, which ranges from 0 (no disability) to 3 (severe disability).

### Statistical analysis

Descriptive statistics were used to summarize the patients’ baseline characteristics. The chi-squared test was used for comparison of proportions. The Mann–Whitney nonparametric test was used for between-group comparisons. Correlations among the SF-36 domains and HAQ-DI scores, clinical parameters and disease activity were investigated by Spearman’s correlation. Spearman’s coefficient values were defined excellent (>0.91), good (0.90-0.71), moderate (0.70-0.51), fair (0.50-0.31), or little or none (<0.30).

## Results

Sixty-six patients with RaP who fulfilled the entry criteria were admitted to the outpatient clinic. The cohort consisted of 63 women and 3 men, aged from 17 to 73 years (median 41 years), with a disease duration from RaP onset ranging from 0.5 to 30 years (median 3.5 years). Thirty-one (46.9%) of them fulfilled the criteria for eSSc and 35 (53.1%) for UCTD. Table 1 shows the main demographic, clinical, laboratory, and capillaroscopic features of the 31 eSSc and 35 UCTD patients. The two groups were similar in terms of age, sex, RaP duration, ANA positivity and prevalence of abnormal routine immune-inflammatory parameters. All patients were taking the maximal tolerated dose of calcium channel blockers (CCB – either nifedipine 20–60 mg/day or amlodipine 5–10 mg/day) to control RaP. By definition, serum SSc-marker autoantibodies (27/31 [87.0%] vs 0/35; p <0.0001) and a capillaroscopic scleroderma pattern (22/31 [70.9%] vs 0/35; p <0.0001) were detected only in eSSc patients. A history of arthritis was found only in UCTD patients (4/35 [11.4%] vs 0/35) (p = 0.052), whereas only 3 patients (all eSSc) had puffy fingers (time of onset from RaP was 2 years in two patients and three years in one patient).

**Table 1:** Demographic, clinical, serological and capillaroscopic data of the eSSc and UCTD patients

**Feature**	**Early SSc**	**UCTD**	**P**
	**n = 31**	**n = 35**	
Sex: F/M (n)	29/2	34/1	ns
Age; years (median; range)	41 (17–64)	40 (18–73)	ns
RaP duration; years (median; range)	3 (1–24)	4 (0.5-30)	ns
Puffy fingers (n;%)	3 (9.6)	0 (0)	ns
History of arthritis (n;%)	0 (0)	4 (11.4)	0.052
ESR >20 mm/h (n;%)	5 (16.1)	3 (8.5)	ns
Gammaglobulins > 1.5 g/dl (n;%)	2 (6.4)	2 (5.7)	ns
C3 < 80 mg/dl or C4 < 15 mg/dl (n;%)	2 (6.4)	3 (8.5)	ns
DLCO < 80% of predicted (n;%)	7 (22.5)	5/20 (25.0)*	ns
E/A ratio < 1** (n;%)	2 (6.4)	1 (2.8)	ns
ANA + (n;%)	30 (96.7)	35 (100)	ns
SSc-marker antibodies + (n;%)	27 (87.0)	0 (0)	-
ACA + (n;%)	20 (64.5)	0 (0)	-
Anti-Scl-70 + (n;%)	6 (19.3)	0 (0)	-
ESSG-AI > 3 (n;%)	0 (0)	-	-
NCM SSc pattern (n;%)	22 (70.9)	0 (0)	-
Calcium channel blockers (n;%)	31 (100)	35 (100)	ns

* In 20 out of 35 UCTD patients the DLCO was measured; **Not otherwise explained.

All data are expressed as numbers and percentages (in brackets), except were otherwise indicated.

*SSc *= systemic sclerosis; *UCTD *= undifferentiated connective tissue disease; *n *= number; *F *= female; *M *= male; *ns *= not statistically significant; *RaP *= Raynaud’s phenomenon; DLCO = carbon monoxide lung diffusion; E/A ratio <1, inverted ratio between early (E)/late (atrial = A) ventricular filling velocity; *ESR *= erythrocyte sedimentation rate; *ANA* = antinuclear antibodies; *ACA *= anticentromere antibodies; *ESSG-AI* = European Scleroderma study group activity index; *NCM *= nailfold capillary microscopy.

The HAQ-DI and SF-36 scores in the two groups of patients and in the controls are summarized in Table 2. Statistically significant differences were found between eSSc and UCTD patients and controls in the scores of the following domains: PCS, MCS, physical functioning, role-physical, bodily pain, general health and mental health. There were no differences in any domain between eSSc and UCTD patients.

**Table 2:** HAQ-DI and SF-36 values in eSSc, UCTD patients and healthy subjects

**SF-36 and HAQ-DI scores**	**eSSc**	**UCTD**	**Controls**	**P1**	**P2**	**P3**
	**n = 31**	**n = 35**	**n = 40**			
PCS (Median; Range)	51 (28–57)	48 (32–58)	59 (46–66)	0.762	**<0.001**	**<0.001**
MCS (Median; Range)	47 (18–67)	48 (25–63)	54 (20–76)	0.943	**0.020**	**0.014**
HAQ-DI (Median; Range)	0 (0–1)	0 (0–1)	0 (0–0.25)	0.572	**0.043**	0.102
Physical functioning (Median; Range)	90 (30–100)	85 (50–100)	100 (90–100)	0.852	**<0.001**	**<0.001**
Role-physical (Median; Range)	100 (0–100)	100 (0–100)	100 (50–100)	0.607	**0.020**	**0.017**
Bodily pain (Median; Range)	64 (22–100)	41 (41–100)	84 (41–100)	0.378	**0.018**	**<0.001**
General health (Median; Range)	61 (10–97)	50 (25–92)	76 (46–100)	0.232	**<0.001**	**<0.001**
Vitality (Median; Range)	65 (15–95)	55 (25–100)	65 (30–100)	0.328	0.145	**0.0071**
Social functioning (Median; Range)	75 (12–100)	75 (25–100)	84 (12–100)	0.210	0.406	0.892
Role-emotional (Median; Range)	66 (0–100)	100 (0–100)	100 (0–100)	0.625	0.314	0.456
Mental health (Median; Range)	68 (8–100)	64 (28–100)	84 (4–100)	0.792	**0.0045**	**0.0011**

*eSSc* = early SSc; *UCTD* = undifferentiated connective tissue disease; *P1* = eSSc vs UCTD; *P2* = eSSc vs controls; *P3* = UCTD vs controls; *PCS* = physical component summary score; *MCS* = mental component summary score; *HAQ-DI* = Health assessment questionnaire–disability index.

At univariate analysis (Tables 3 and 4), PCS was negatively correlated to HAQ-DI (rho −0.59; p = 0.0004) and ESR >20 mm/h (rho −0.58; p = 0.0006) in eSSc patients. No statistically significant correlation was found between PCS, MCS and HAQ-DI in UCTD patients. Age, sex, disease duration, history of arthritis, low levels of either C3 or C4, a low DLCO (carbon monoxide lung diffusion) and inversion of the E/A ratio were not correlated to PCS and MCS in eSSc or UCTD patients. The disease duration in 5 UCTD patients was shorter than 3 years. Considering these patients as pre-CTD (connective tissue disease) and excluding them from the analysis, the same results were obtained.

**Table 3:** Correlations among clinic and laboratory features and PCS and MCS scores in eSSc patients

**Clinical and laboratory features**	**PCS (rho; P)**	**MCS (rho; P)**
Age	−0.15 (0.413)	0.481 (0.060)
Sex F	−0.19 (0.283)	0.007 (0.968)
Disease duration	−0.19 (0.281)	0.0049 (0.979)
History of arthritis	0	0
ESR >20 mm/h	**−0.58 (0.0006)**	−0.049 (0.793)
Gammaglobulins > 1.5 g/dl	−0.06 (0.723)	0.117 (0.528)
C3 < 80 mg/dl or C4 < 15 mg/dl	0.133 (0.475)	0.112 (0.547)
Low DLCO (<80% of predicted)	0.056 (0.763)	0.142 (0.444)
E/A ration < 1	−0.09 (0.607)	0.027 (0.883)
HAQ-DI	**−0.59 (0.0004)**	−0.298 (0.103)
Active disease	0	0

*PCS* = physical component summary score; *MCS* = mental component summary score; *ESR* = erythrocyte sedimentation rate; DLCO = carbon monoxide lung diffusion; E/A ratio <1, inverted ratio between early (E)/late (atrial = A) ventricular filling velocity; *HAQ-DI* = Health assessment questionnaire–disability index.

**Table 4:** Correlations among clinic and laboratory features and PCS and MCS scores in UCTD patients

**Clinical and laboratory features**	**PCS (rho; P)**	**MCS (rho; P)**
Age	−0.03 (0.831)	0.08 (0.607)
Sex F	−0.02 (0.883)	0.223 (0.201)
Disease duration	0.166 (0.339)	0.199 (0.246)
History of arthritis	0.104 (0.561)	0.136 (0.433)
ESR >20 mm/h	−0.290 (0.085)	0.303 (0.070)
Gammaglobulins > 1.5 g/dl	−0.231 (0.823)	−0.178 (0.623)
C3 < 80 mg/dl or C4 < 15 mg/dl	−0.176 (0.454)	−0.234 (0.234)
Low DLCO (<80% of predicted)	−0.08 (0.720)	0.185 (0.446)
E/A ratio < 1	−0.10 (0.561)	0.061 (0.728)
HAQ-DI	−0.44 (0.070)	−0.060 (0.725)

*PCS* = physical component summary score; *MCS* = mental component summary score; *ESR* = erythrocyte sedimentation rate; DLCO = carbon monoxide lung diffusion; E/A ratio <1, inverted ratio between early (E)/late (atrial = A) ventricular filling velocity; *HAQ-DI* = Health assessment questionnaire–disability index.

## Discussion

This study was undertaken to assess the quality of life in eSSc patients compared to that in UCTD and controls. We found that HRQOL was impaired in both eSSc and UCTD patients.

It would have been interesting to assess HRQOL in patients with primary RaP, in whom an impaired HRQOL has been recently pointed out by either SF-36 or EUROQoL 5 item questionnaire (EQ-5D) [20]. However, few patients with such a condition are referred to our Rheumatologic tertiary centre.

Our results show that eSSc patients, who, by definition, experience only Raynaud’s phenomenon but no other SSc-specific clinical manifestation, perceive an impaired physical and mental quality of life, as happens in other subgroups of SSc patients [7,21]. Here we show that disability and an increase in ESR levels are negatively associated with physical health status. In these patients the only functional impairment was the RaP and/or the presence of puffy hands (this latter condition in only 3 eSSc patients).

Since HRQOL has been reported to be impaired in patients with primary RaP, one could postulate that the impairment in HRQOL in SSc and UCTD patients might be ascribed to RaP itself. Such assumption, however, would not totally justify this condition, especially considering that these patients were taking the maximum tolerated dose of vasodilators prescribed to decrease the frequency and duration of RaP attacks and that most of them stated to have a good control of their RaP condition.

Given the low burden of disability registered, we can postulate that the clinical measures commonly assessed in eSSc and UCTD do not completely account for the poor quality of life perceived by these patients and this could be explained in different ways.

First of all, a great difference between the assessment of the disease severity and HRQOL has to be taken into account: in fact, the former is made through a pre-specified combination of clinical, laboratory and instrumental exams which might overlook some aspects of the disease influencing the health status, that is investigated, as known, by self-report questionnaires.

Moreover, a recent study of HRQOL in SSc patients suggested that the most important determinants of physical and mental health are the patient’s cognitive representations of the illness [22]. Arat et al. [22] observed that the fear of clinical consequences and the tendency to attribute each physical complaint to SSc (“illness identity”), are the main contributors to physical health, whereas the emotional responses to personal representation of the disease are the main contributors to mental health. In addition to illness perception, the use of various strategies to cope better with the disease is related to physical and mental health [22].

Given the above observations, it is conceivable that in eSSc and UCTD patients similar psychological mechanisms might contribute, together with the disease –related symptoms to the worse quality of life. Unfortunately, one of the shortcomings of our study is that we did not include instruments that measure psychosocial factors, and so no conclusion about the relationships between psychosocial factors and HRQOL can be drawn.

ANA positivity in patients with RaP has long been known to be a factor predictive of the development of a CTD [23,24]. In that regard, the absence of any significant difference between patients with (eSSc) and without (UCTD) marker autoantibody would suggest that the presence of an autoimmune RaP might be sufficient to affect HRQOL.

This study has some limitations. First of all, the number of patients enrolled is relatively low so that a larger sample is required to confirm these results. Moreover, as already stated it would have been intriguing to assess the HRQOL also in patients affected by a primary RaP, but unfortunately few patients with such a condition are referred to our centre. Finally, our data do not allow us to identify other determinants of the reduced quality of life in both mental and physical domains, apart from those described.

In conclusion, we assessed HRQOL in eSSc and UCTD patients and looked for associations between clinical features and health status perception. Although the correlations observed do not reflect a causal relationship, our results might be useful to identify targets of intervention. Our data would suggest that also these patients experience an impaired quality of life in both physical and mental domains. This condition has to be taken into account by the clinicians involved in their care. In the absence of a therapy able to definitively block injury to lungs, joints and other internal organs, further studies are needed to completely identify the factors that contribute to the poor health status in eSSc and UCTD patients.

## Abbreviations

SSc: Systemic sclerosis; HRQOL: Health-related quality of life; SF-36: Short Form-36; eSSc: Early systemic sclerosis; UCTD: Undifferentiated connective tissue disease; RaP: Raynaud’s phenomenon; ANA: Antinuclear antibodies; BUN: Blood urea nitrogen; ALT: Alanine transaminase; AST: Aspartate transaminase; ESR: Erythrocyte sedimentation rate; NVC: Nailfold videocapillaroscopy; HAQ-DI: Health assessment questionnaire disability index; PCS: Physical component score; MCS: Mental component score; CTD: Connective tissue disease.

## Competing interests

The authors declare that they have no competing interests.

## Authors’ contribution

MI, GC, SV, MA and GV: substantial contribution to design the study, to collect data ant to draft the manuscript. All authors have given final approval of the submitted version.
